# Supplementary material for: Inhibition of the serine protease HtrA1 by SerpinE2 suggests an extracellular proteolytic pathway in the control of neural crest migration
Source: eLife. 2024 Apr 18;12:RP91864. doi: 10.7554/eLife.91864 (PMC11026092; doi:10.7554/eLife.91864)
Supplement: Figure 6—figure supplement 2—source data 1. [file elife-91864-fig6-figsupp2-data1.pdf]

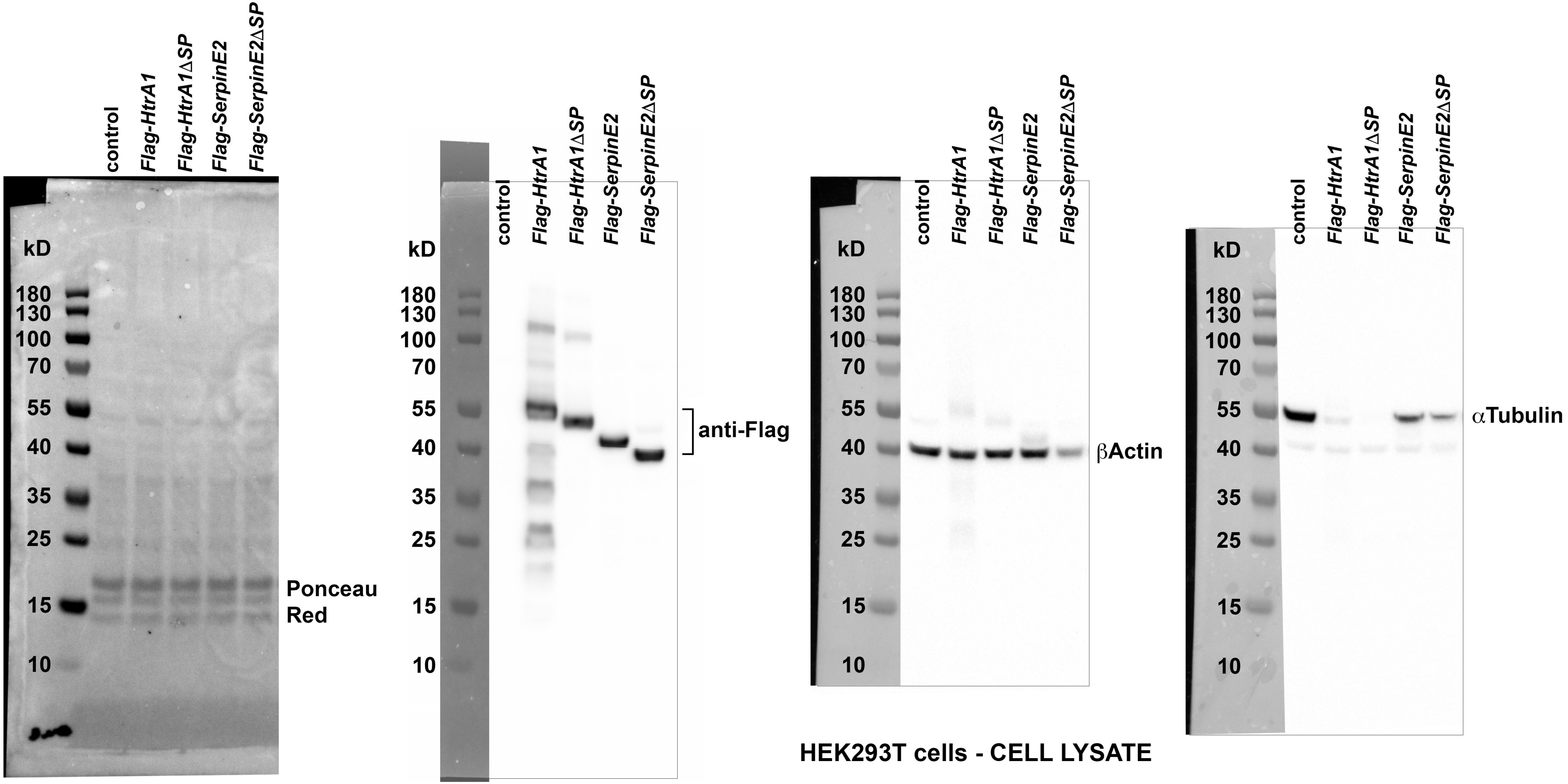

HEK293T cells - CELL LYSATE
