# Supplementary figures and images for: Inhibition of the serine protease HtrA1 by SerpinE2 suggests an extracellular proteolytic pathway in the control of neural crest migration
Source: eLife. 2024 Apr 18;12:RP91864. doi: 10.7554/eLife.91864 (PMC11026092; doi:10.7554/eLife.91864)

# HEK293T cells - SUPERNATANT

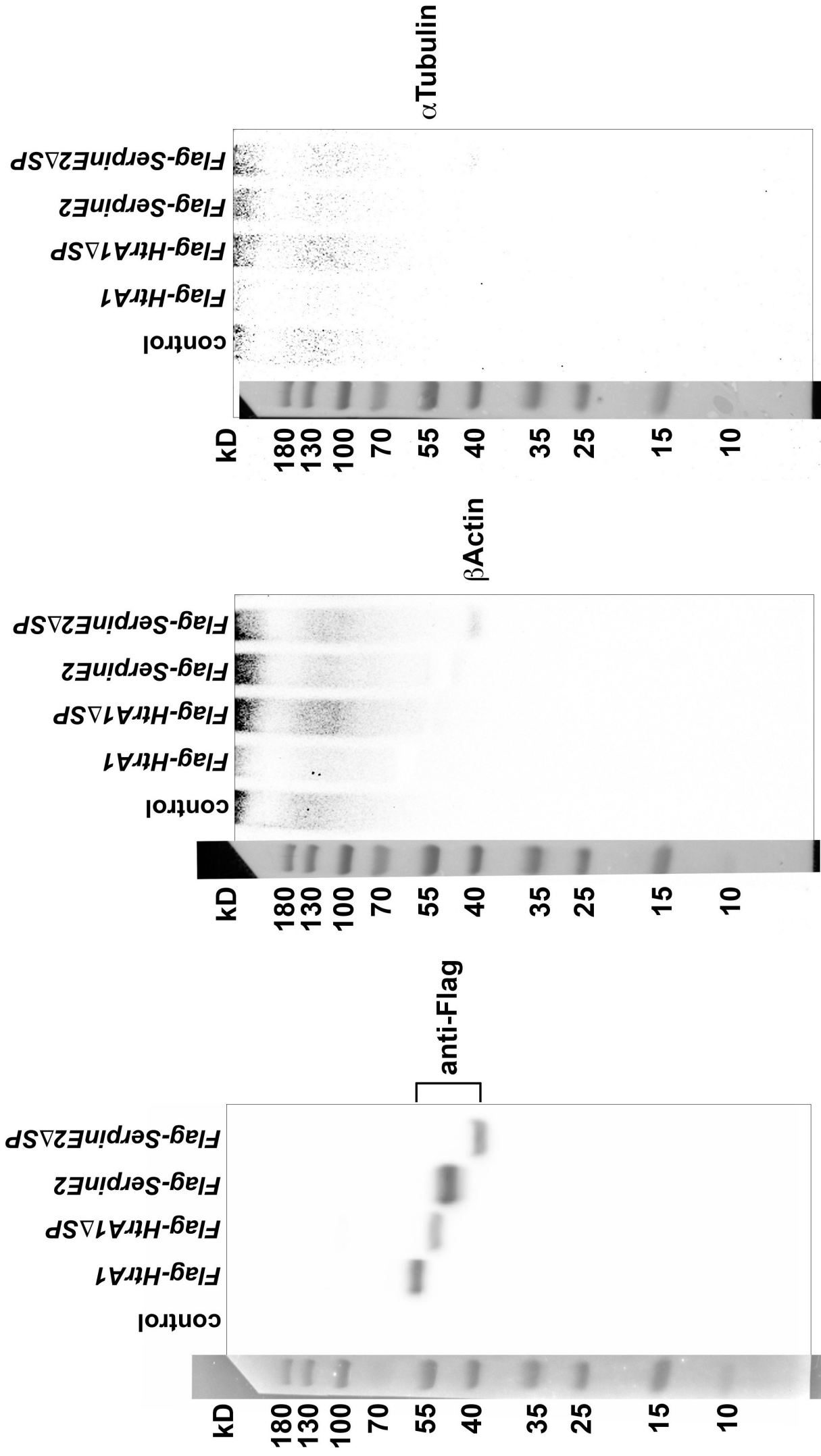

Supplement: Figure 6—figure supplement 2—source data 2. [file elife-91864-fig6-figsupp2-data2.pdf]

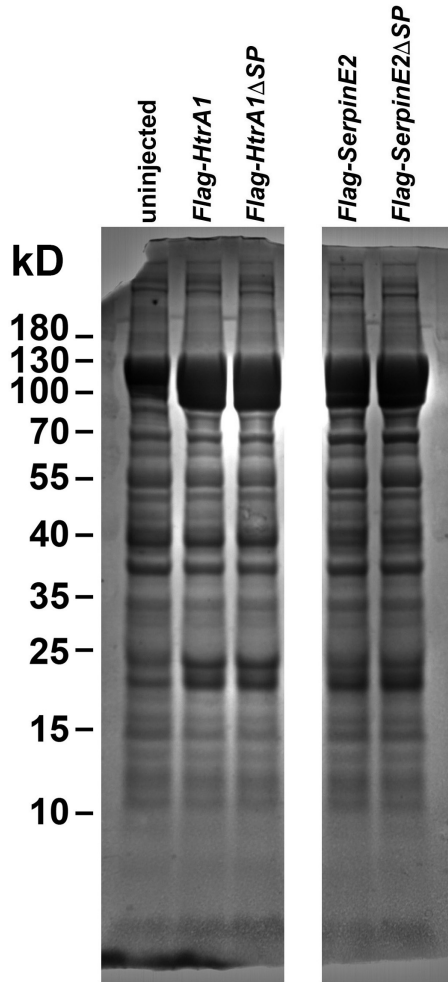

Coomassie-Blue (Gel)

*Xenopus* embryos (stage 10.5)

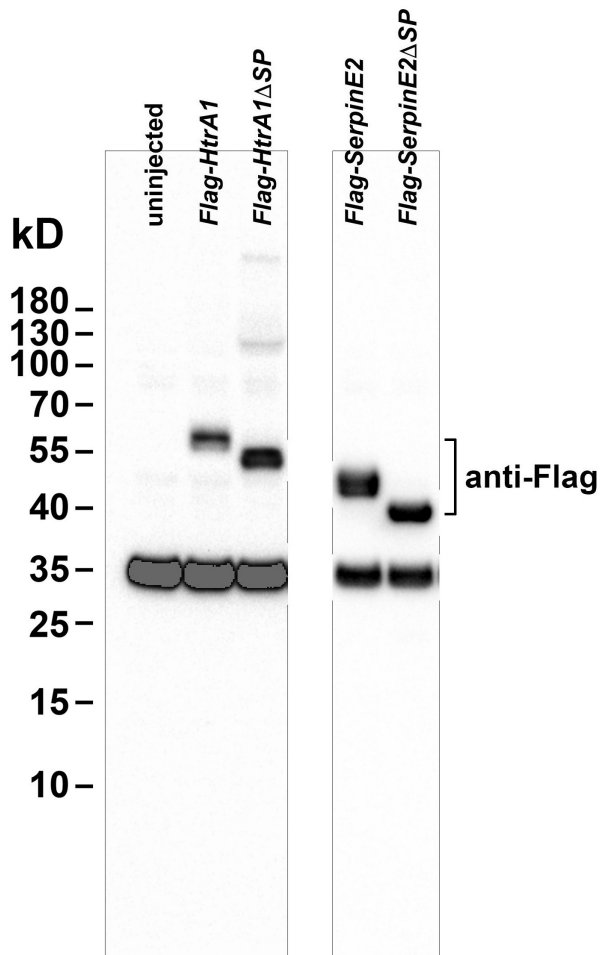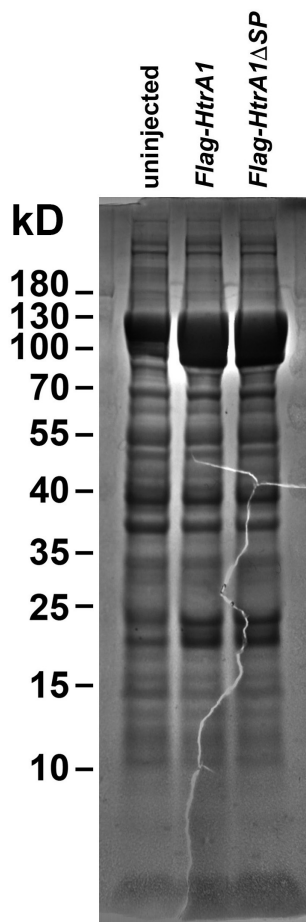

Coomassie-Blue (Gel)

*Xenopus* embryos (stage 10.5)

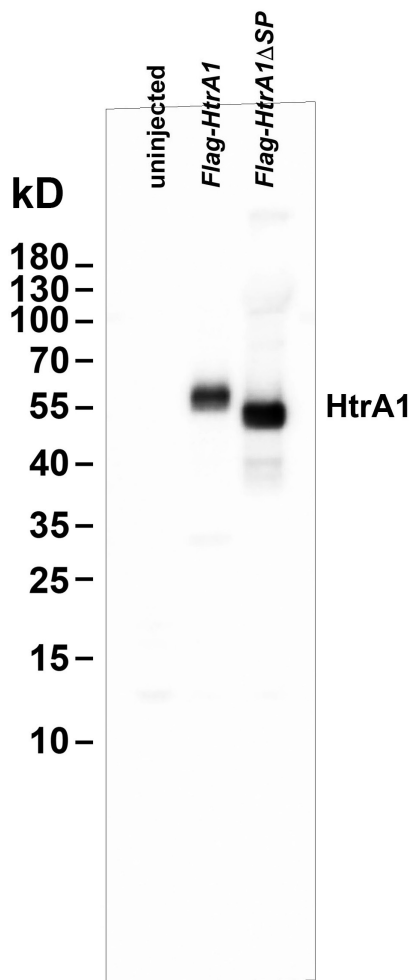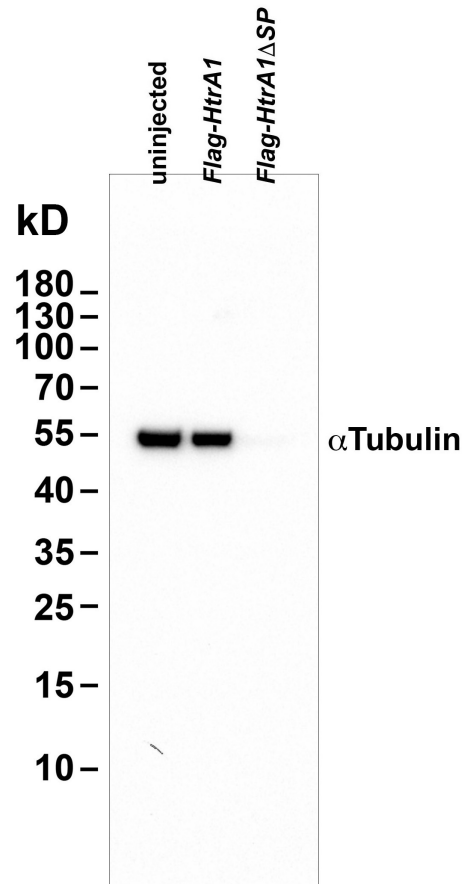

Supplement: Figure 6—figure supplement 2—source data 3. [file elife-91864-fig6-figsupp2-data3.pdf]
